# Towards Treating Multiple Sclerosis Progression

**DOI:** 10.3390/ph17111474

**Published:** 2024-11-02

**Authors:** Darius Häusler, Martin S. Weber

**Affiliations:** 1 Institute of Neuropathology, University Medical Centre, 37075 Goettingen, Germany; darius.haeusler@med.uni-goettingen.de; 2 Fraunhofer-Institute for Translational Medicine and Pharmacology ITMP, 37075 Goettingen, Germany; 3 Department of Neurology, University Medical Centre, 37075 Goettingen, Germany

**Keywords:** multiple sclerosis, progression, treatment, therapy, neuroprotection, remyelination, neurorepair, regeneration, compartmentalized inflammation, clinical trials

## Abstract

Multiple sclerosis (MS) is an inflammatory, demyelinating disease of the central nervous system (CNS). In most patients, the disease starts with an acute onset followed by a remission phase, subsequent relapses and a later transition to steady chronic progression. In a minority of patients, this progressive phase develops from the beginning. MS relapses are characterized predominantly by the de novo formation of an inflammatory CNS lesion and the infiltration of immune cells, whereas the pathological features of MS progression include slowly expanding lesions, global brain atrophy and an inflammatory response predominantly mediated by macrophages/microglia. Importantly, this CNS-intrinsic pathophysiology appears to initiate early during the relapsing–remitting disease phase, while it turns into the key clinical MS feature in later stages. Currently approved disease-modifying treatments for MS are effective in modulating peripheral immunity by dampening immune cell activity or preventing the migration of immune cells into the CNS, resulting in the prevention of relapses; however, they show limited success in halting MS progression. In this manuscript, we first describe the pathological mechanisms of MS and summarize the approved therapeutics for MS progression. We also review the treatment options for progressive MS (PMS) that are currently under investigation. Finally, we discuss potential targets for novel treatment strategies in PMS.

## 1. Introduction

MS is the most common autoimmune disease of the CNS, affecting around 2.8 million people worldwide. The first clinical symptoms typically appear between the ages of 20 and 40 and affect twice as many women as men [1]. Most patients experience an acute onset of the disease followed by a period of remission [2]. After relapses, the disease progresses to a chronic course, while a small percentage of patients develop this progressive phase from the outset. MS relapses are characterized by the activation and migration of peripheral immune cells into the CNS, leading to perivascular inflammatory lesions [3]. In contrast, the progression of MS without relapses is mainly driven by compartmentalized inflammation, in which T cells, B cells and myeloid cells are trapped in the brain and spinal cord behind a closed blood-brain barrier (BBB), and by degenerative disease mechanisms [4]. Several lines of evidence suggest that insidious progression occurs in the earliest phases of the disease [5,6,7] and becomes the main clinical feature of MS in later stages [4].

Over the past few decades, tremendous progress has been made in the development of disease-modifying therapies (DMTs) for the treatment of relapsing-remitting MS (RRMS). Although therapeutic options in patients with PMS are limited, recent research has led to the validation of new promising treatment strategies in clinical trials with PMS patients.

In this manuscript, we first describe the pathological mechanisms involved in disease progression. Furthermore, we summarize the current knowledge about siponimod and ocrelizumab, which are approved drugs for the treatment of secondary progressive MS (SPMS), while ocrelizumab is currently the only drug approved for the treatment of primary progressive MS (PPMS). Moreover, we provide an overview of the most promising therapeutics currently under investigation for the treatment of PMS. Finally, we discuss potential targets for novel treatment strategies in PMS.

## 2. MS Pathology and Pathogenesis–PMS

The pathological hallmark of RRMS is the presence of focal inflammatory lesions in the white matter, while the pathologic process in PMS is assumed to be more complex, consisting of a variety of different mechanisms and pathways. Besides neurodegeneration, this also includes inflammation, which takes place behind an intact BBB. This so-called compartmentalized inflammation consists of B cell, T cell and myeloid cell aggregates in the meninges and perivascular cuffs [8,9,10]. These meningeal cell aggregates secrete cytokines, free radicals and proteases [11,12] and are assumed to have important roles in cortical gray matter damage as they are associated with elevated subpial demyelination, a higher level of cortical lesion formation and increased neuronal degeneration [9,13]. Microglia and macrophages have a central role in the pathomechanisms of chronic active lesions, which are the most common in PMS patients and are also referred to as smoldering lesions [14,15]. Numerous activated, iron-positive microglia and macrophages can be found at the edges of slowly expanding lesions (SELs), a subgroup of chronic white matter lesions, which are suggested to promote lesion expansion [16]. Moreover, a correlation between activated microglia/macrophages and the extent of paranodal disruption, ongoing demyelination and neuroaxonal injury has been made, indicating a crucial role in neurodegeneration [17,18]. This process of neurodegeneration can be directly induced by microglia and macrophages upon the release of neurotoxic factors [11,19,20]. Activated microglia and macrophages can also release reactive oxygen species (ROS) and reactive nitrogen species (RNS), which are known to induce mitochondrial dysfunction and injury to cells, leading to the degeneration of oligodendrocytes and neurons [21]. Iron is known to be an essential cofactor in myelin formation by oligodendrocytes [22,23]. CNS inflammation can promote extracellular iron accumulation [24], resulting in cell injury through protein misfolding, glutamate excitotoxicity, oxidative stress and ferroptosis [25]. Remyelination is the natural repair mechanism of demyelination, showing its effectiveness in the early stages of disease, while it declines over time [26]. Moreover, it has been shown that the proportion of remyelinated lesions is lower in PMS than in RRMS [10]. This ineffective remyelination in PMS is attributed to persistent neuroinflammation, the loss of oligodendrocyte lineage cells and the pathogenic microenvironment [27,28,29].

## 3. Approved Therapeutics for MS Progression

Since the release of IFN β-1b in 1993, as the first approved disease-modifying treatment (DMT) for MS, many other drugs have been successfully developed for the treatment of RRMS. However, the use of these approved DMTs in progressive MS has shown largely disappointing results. The first DMT approved in Western Europe and North America for the use of SPMS was mitoxantrone in 2000 based on the MIMS trial [30]. Mitoxantrone showed positive effects on the relapse rate and the progression of disability, but it is currently no longer used in many countries due to its severe side effects [31]. Cladribine, a DNA synthesis inhibitor, which selectively diminishes lymphocyte numbers in the periphery by the induction of apoptosis, was also approved for the treatment of active SPMS by the FDA in 2019 based on the CLARITY clinical trial (NCT00213135). A post hoc analysis showed that patients with relapsing MS had a lower risk of progression to SPMS within two years of treatment [32]. Although cladribine is able to cross the BBB [33] and in vitro data have shown microglia inhibition upon treatment in culture [34], it is questionable whether the brief drug administration regimen is able to cause long-lasting therapeutic effects on the CNS. Siponimod and ocrelizumab are two other therapeutic agents that are available for the treatment of PMS and whose properties we describe in more detail below.

### 3.1. Siponimod

Siponimod is a selective sphingosine-1-phosphate (S1P)-1 and S1P-5 modulator that is not only able to reduce the number of circulating lymphocytes by preventing their egress from the lymph nodes but can also pass through the BBB due to its small size and lipophilic nature [35]. Both target receptors can be found on CNS-resident cells, enabling siponimod to exert central effects in CNS autoimmune diseases [36]. In various animal models of MS, siponimod decreased meningeal inflammation and underlying cortical demyelination [37], attenuated microgliosis and astrogliosis [38,39], reduced oxidative stress and mitochondrial injury [37] and induced remyelination [40]. The phase III EXPAND trial investigated the efficacy of siponimod in 1651 patients. Siponimod reduced the risk of 3-month confirmed disability progression by 21% compared with placebo [41]. Moreover, the increase in T2 lesion volume from baseline was lower, and the brain volume was preserved to a higher degree in siponimod-treated patients, suggesting neuroprotective effects. Based on these findings, the FDA approved the use of siponimod for the treatment of SPMS in patients with active disease in 2019.

### 3.2. Ocrelizumab

B cells play an important pathogenic role in MS, and a substantial reduction in new lesion formation as well as the annual relapse rate could be shown in randomized clinical trials in RRMS using the monoclonal anti-CD20 antibodies rituximab and ocrelizumab [42,43]. With the OLYMPUS trial, rituximab was the first B cell-depleting therapy tested for its efficacy in PMS [44]. Although the OLYMPUS trial reported negative results, a subgroup analysis revealed reduced disease progression in patients younger than 52 years of age with active inflammatory lesions. The findings from the OLYMPUS trial had a significant impact on the design of the ORATORIO trial, the second study comparing ocrelizumab versus placebo in 732 PMS patients [45]. The inclusion criteria for this study were patients younger than 55 years of age, an Expanded Disability Status Scale (EDSS) score of 3.0 to 6.5 and a positive IgG index or evidence of cerebrospinal fluid (CSF)-specific oligoclonal bands. The primary endpoint of this study was met, showing lower rates of 12-week confirmed disability progression in ocrelizumab-treated patients as compared to placebo, with a relative risk reduction of 24%. Based on the positive results of the ORATORIO study, ocrelizumab was approved by the FDA and remains the only DMT approved for PPMS. To better understand the mechanism of action of ocrelizumab and the biology of B cells in RMS or PPMS, an open-label, multicenter biomarker study was conducted (NCT02688985). In this study, the prognosis of nonrelapsing progressive disease was associated with neurofilament heavy-chain and activated glial markers [46]. In addition, a correlation was found between the long-term progression of multiple sclerosis and increases in glial fibrillary acidic protein in the CSF, suggesting that the biology of nonrelapsing progressive MS is related to insidious damage from neurodegeneration and/or the smoldering compartmentalized inflammation of the CNS [15]. In contrast to its beneficial use in young and active PMS patients, anti-CD20 treatment is therefore thought to be less effective in preventing clinical progression independent of relapse activity (PIRA), a process that could be linked to both smoldering inflammation and neurodegeneration [7]. An ongoing multicenter, randomized, double-blind, controlled phase III study is currently evaluating the efficacy, safety and pharmacokinetics of double or triple doses of ocrelizumab in adults with PPMS compared to the approved 600 mg dose (NCT04548999).

## 4. Therapeutics under Investigation for MS Progression

Several therapeutics for the treatment of PMS are currently being evaluated in advanced clinical trials (Table 1). Besides the modulation of CNS-compartmentalized inflammation, many of these therapeutics target alternative pathophysiological pathways to achieve neuroprotection and/or remyelination. In this section, we present some of the most promising candidates.

### 4.1. Bruton’s Tyrosine Kinase Inhibitors

Bruton’s tyrosine kinase (BTK) is a non-receptor, cytoplasmic protein tyrosine kinase expressed on most hematopoietic cell populations including B cells and myeloid cells [47]. In B cells, BTK is an essential component of multiple signaling pathways downstream of the B cell receptor controlling B cell survival, activation, maturation, cytokine production and the antigen-dependent stimulation of T lymphocytes [48]. In myeloid cells such as macrophages and microglia, BTK is involved in Fc receptor signaling regulating activation, cytokine secretion, ROS production and inflammasome activation [49]. Since BTK inhibition is an effective therapeutic strategy for the treatment of many B cell lymphomas, interest has grown in testing its efficacy in the treatment of MS, in which B cells play an important pathogenic role [48]. In the experimental autoimmune encephalomyelitis (EAE) model of MS, the BTK inhibitor evobrutinib diminished the antigen-dependent activation and maturation of B cells and decreased the release of pro-inflammatory cytokines [50]. In addition, evobrutinib reduced the ability of B cells to act as antigen-presenting cells in the development of encephalitogenic T cells, resulting in decreased CNS infiltration and clinical severity. Due to the molecular size and pharmacological properties, it is assumed that BTK inhibitors are able to cross the BBB [51] but with varying degrees of effectiveness [52]. Accordingly, BTK inhibitors provide an opportunity to modulate not only the peripheral immune system but through the BBB penetrance also CNS-compartmentalized B cell and myeloid cell inflammation, a hallmark of PMS. In a recent study of our group, the BTK inhibitor evobrutinib reduced the pro-inflammatory activity of microglia in the EAE model of MS [53]. Moreover, in a model of toxic demyelination induced by cuprizone, evobrutinib promoted the clearance of myelin debris by microglia, resulting in an accelerated remyelination. These findings indicate that BTK inhibition might be a promising therapeutic approach in targeting CNS-compartmentalized inflammation, leading to a decreased disease progression. The two BTK inhibitors tolebrutinib and fenebrutinib are currently being analyzed for their potential effects on PMS, which we discuss in more detail below.

### 4.2. Tolebrutinib

The covalent and irreversible BTK inhibitor tolebrutinib is being evaluated in two phase III trials, HERCULES in SPMS (NCT04411641) and PERSEUS in PPMS (NCT04458051). Both studies are double-blind, randomized, placebo-controlled trials and aim to investigate the effects of treatment on delaying the progression of disability. In the HERCULES trial, the efficacy and safety of tolebrutinib were investigated in 1131 patients with nonrelapsing SPMS, defined as no clinical relapses for the past 24 months, documented evidence of disability accumulation in the past 12 months and an EDSS score between 3.0 and 6.5. The study was recently completed and met its primary endpoint by demonstrating a 31% risk reduction in time to confirmed disability progression at 6 months in MS patients treated with tolebrutinib compared to placebo [54]. In addition, key secondary endpoints were met, including an increase in the probability of achieving confirmed improvement in disability at 6 months and a reduction in the annualized rate of new/enlarging T2 lesions. Of note, similar to evobrutinib in two phase III trials in RRMS (NCT04338022 and NCT04338061), tolebrutinib failed to meet the primary endpoint of reducing the annualized adjudicated relapse rate in two recent phase III trials (NCT04410978 and NCT04410991) in RRMS compared to teriflunomide [55]. Although the analysis of secondary endpoints revealed a 29% risk reduction in 6-month CDP, the number of new Gd-enhancing T1 lesions was higher in one treatment group receiving tolebrutinib than the one receiving teriflunomide. These results suggest that acute focal inflammation and smoldering neuroinflammation are two distinct biological processes and that BTKi would be much more suitable for the treatment of PMS than relapsing prophylaxis. Thus, tolebrutinib represents a breakthrough and may be the first therapy of its kind to offer clinically meaningful improvement in disability progression. It remains to be seen whether tolebrutinib can show equally positive results in the second trial, which is recruiting PPMS patients and is scheduled to end in July 2025.

### 4.3. Fenebrutinib

The non-covalent and reversible BTK inhibitor fenebrutinib is being evaluated for safety and efficacy in disease progression in patients with PPMS in the FENtrepid trial (NCT04544449). A total of 985 participants were enrolled in this double-blind, randomized study, and the treatment effects of fenebrutinib are being compared with those of ocrelizumab, the only approved drug for the treatment of PPMS to date [56]. The primary outcome is the time to onset of composite 12-week confirmed disability progression, and the study is expected to be completed by the end of 2026.

### 4.4. Metformin

Metformin is a synthetic guanidine derivative that has a blood glucose-lowering effect [57] and is a first-line drug in the treatment of type 2 diabetes mellitus, especially in patients with obesity [58]. In addition to its blood glucose-lowering effect, metformin also has anti-inflammatory and antioxidant properties, which are attributed to the activation of adenosine monophosphate-activated protein kinase (AMPK) [59,60]. In EAE, metformin decreased immune cell infiltration and reduced the expression of pro-inflammatory cytokines, cell adhesion molecules, matrix metalloproteinase 9 and the chemokine RANTES in the CNS of EAE animals, resulting in the slowing of disease progression [61]. In the cuprizone model, metformin treatment reduced cuprizone-induced NF-κB activation by upregulating AMPK activity, downregulated the expression of pro-inflammatory microglia-associated genes, decreased inducible NO synthase (iNOS) protein levels, and increased the anti-inflammatory protein Trem2 in the corpus callosum [62]. Moreover, further studies have shown that metformin reduces endogenous ROS levels and associated DNA damage [63] and improves the remyelination capacity of aged human OPCs by restoring their responsiveness to pro-differentiation signals [64]. Based on its ability to cross the BBB [65] and exert antioxidant, neuroprotective and regulatory effects on neuroinflammation and microglia activity, metformin may be a suitable candidate for the treatment of PMS. Little is known so far about metformin treatment’s effects on MS patients. In a prospective cohort study including a total of 50 obese RRMS patients with metabolic syndrome, treatment with metformin led to a reduction in the number of new or enlarging T2 brain lesions on MRI in 20 patients [66]. In addition, the study demonstrated an increased level of AMPK expression as well as an elevated proportion of Treg cells and a decreased production of IFN-γ and IL-17 in MS patients treated with metformin. Further studies of metformin in the treatment of PMS are currently being prepared. In the USA, for example, the recruitment of patients for a randomized, placebo-controlled phase I clinical trial in PMS patients has recently begun (NCT05349474). The safety and efficacy of the metformin add-on treatment will be investigated in an estimated 44 PMS patients for 12 months until the end of the study in mid-2026. A second clinical trial is currently recruiting 120 patients for a multicenter, randomized, placebo-controlled phase II clinical trial in PMS patients (NCT05893225). The aim of this clinical trial is to demonstrate the neuroprotective and remyelinating effects of metformin in MS patients. The primary endpoint is the change in walking speed as measured by the 25-foot walk test (T25FW) between baseline and 96 weeks of treatment. The study is scheduled to be finished by the end of 2026. Another clinical trial is a large multicenter, multi-arm, multi-stage, randomized, placebo-controlled phase II/III clinical trial (OCTOPUS) currently recruiting 1200 patients (ISRCTN14048364). The aim of this clinical trial is to evaluate the safety and efficacy of several drugs, including metformin, in both SPMS and PPMS patients. The primary endpoint is the whole brain atrophy rate as measured by MRI and disability progression as measured by a multicomponent measure of disability progression that includes the EDSS, the T25FW and the 9-hole peg test (9HPT). The study is scheduled to be completed by the end of 2028.

### 4.5. Ibudilast

Ibudilast is an inhibitor for several cyclic nucleotide phosphodiesterases, toll-like receptor 4 and macrophage migration inhibitory factor [67]. In vitro, ibudilast decreased pro-inflammatory cytokine production such as that of tumor necrosis factor α (TNF-α), interleukin-1b (IL-1b) and IL-6; increased the secretion of the anti-inflammatory cytokine IL-10 as well as several neurotrophic factors by activated microglia [68,69] and inhibited the microglia-mediated destruction of neurons [70]. In addition, ibrutinib reduced spinal cord inflammation and improved clinical severity in EAE [71]. Due to its small size, ibrutinib is known to be able to cross the BBB [72], suggesting a positive effect on compartmentalized inflammation and neuroprotective properties. Ibudilast was tested in the double-blind, randomized and placebo-controlled phase II SPRINT-MS trial (NCT01982942), which included a total of 255 patients with a similar number of SPMS and PPMS participants [73]. The primary endpoint was met, which was defined as a reduction in MRI-derived brain atrophy over 96 weeks. Post hoc analyses showed that the treatment effect was largely limited to people with PPMS and not SPMS, which may be related to the faster progression of brain atrophy in the PPMS placebo group than that in the SPMS placebo group [74]. Although ibudilast showed no effects on new or enlarging T1 and T2 lesions [75] and did not reduce neurofilament levels in serum or CSF [76], ibudilast was significantly associated with smaller SEL volumes, which were reduced by 23% [77]. In addition, ibudilast had a significant effect on reducing MRI changes in SLEs over time, suggesting a reduction in chronic active lesions and a treatment effect on compartmentalized inflammation. The effect of ibudilast on SELs in combination with the observed effect on brain atrophy make this therapeutic agent attractive for a phase III trial in PMS.

### 4.6. Vidofludimus Calcium (IMU-838)

Vidofludimus calcium is a small molecule that is currently being developed as an oral treatment option for MS or other chronic inflammatory and autoimmune diseases [78]. Similar to teriflunomide, which is already approved for MS, vidofludimus calcium inhibits the enzyme dihydroorotate dehydrogenase (DHODH) in the mitochondria and thus prevents the de novo synthesis of pyrimidine in activated lymphocytes, leading to altered cell proliferation and apoptosis [79]. In addition, vidofludimus calcium also activates the transcription factor Nuclear Receptor-Related 1 (Nurr1), which is mainly expressed in neurons, astrocytes and microglia and is associated with direct neuroprotective properties [80,81], suggesting it as a suitable candidate for the treatment of PMS. In vitro, vidofludimus calcium reduced the neurotoxic environment in human microglia as well as primary monocytes and protected neurons under pro-apoptotic conditions [82]. In EAE, vidofludimus calcium attenuated the severity of the disease and increased the expression of Nurr1 target genes in the CNS [82]. Vidofludimus calcium is currently being evaluated for efficacy, safety and tolerability in a multicenter, randomized, double-blind, placebo-controlled phase II CALLIPER trial in 467 patients with PPMS and SPMS who will be followed for up to 120 weeks (NCT05054140). The primary endpoint is the annualized rate of percent change in brain volume during the main treatment period, and the study is expected to be completed by early 2025. A pre-planned interim analysis to assess serum neurofilament light chain (NfL) was performed after half of the study participants had completed the 24-week study treatment [83]. In 203 patients, composed of 61% nonactive SPMS, 10% active SPMS and 29% PPMS patients, the average serum Nfl in the vidofludimus calcium group decreased by 22.4% from baseline to week 24 compared to placebo. These results support the potential efficacy of vidofludimus calcium in slowing disease progression in PMS. 

### 4.7. Hydroxychloroquine

Hydroxychloroquine (HCQ) is approved for the treatment or prophylaxis of malaria and for the treatment of autoimmune diseases such as chronic discoid lupus erythematosus, systemic lupus erythematosus in adults and rheumatoid arthritis [84]. HCQ is a small molecule and has been shown to cross the BBB [85]. In vitro studies have shown that HCQ inhibits the activity of human microglia and B cells and improves the iron-induced destruction of neurons [86,87]. In EAE, pretreatment with HCQ delayed the onset of the disease, reduced the number of IBA-1-positive microglia/macrophages and decreased white matter demyelination [86]. The combined use of HCQ with the anti-hypertensive drug indapamide, which scavenges oxygen radicals and protects neurons from iron-induced death, resulted in less oxidative and neuroaxonal damage in the lysolecithin model of spinal cord demyelination [88]. In a recently completed open-label, single-center, single-arm futility phase II trial, HCQ was analyzed to reduce the progression of disability in 35 patients with PPMS (NCT02913157). The primary endpoint was the change in T25FW performance between 6 and 18 months of follow-up. Disease worsening occurred in 8 of the 35 participants, and based on the null hypothesis, the researchers concluded that treatment with HCQ results in less disability worsening in PPMS and should be further investigated in randomized controlled clinical trials [89].

## 5. Target Identification for Novel Treatment Strategies in PMS

Although drug development for PMS has increased significantly in recent years, there is still a need to find new therapeutic targets for the disease progression of MS. Direct therapeutic neuroprotection and/or remyelination remains an important research focus, even though several recent studies have shown negative results (lipoic acid, NCT03161028 [90]; simvastatin, NCT03387670 [91]). 

The BBB-penetrating small molecule **Sephin-1**, a selective inhibitor of holophosphatase [92,93], has been shown to prolong eIF2a phosphorylation in stressed primary oligodendrocyte cultures, leading to an increased synthesis of protective proteins and higher oligodendrocyte survival [94]. In addition, Sephin1 delayed the onset of clinical symptoms in EAE, which was correlated with a prolonged integrated stress response, reduced oligodendrocyte and axon loss and diminished T cell numbers in the CNS. Thus, neuroprotective treatment based on improving the integrated stress response could have positive effects on PMS. 

Another strategy to promote neuroprotection and remyelination in PMS is the use of endogenous metabolites. **Taurine** is an ubiquitous, non-proteinaceous amino acid present in the brain in millimolar concentrations [95] and is known as an osmoregulator and neuromodulator [95,96]. Taurine is involved in the modulation of neuronal excitability, antioxidation [97] and, as recently shown, in the differentiation of oligodendrocytes [98]. As a drug, taurine is very well tolerated and is actively transported through the BBB [95]. Therefore, taurine supplementation could be a promising treatment strategy to improve neuroprotection and remyelination. 

Another approach to promote the protection of neurons and oligodendrocytes in PMS could be to target novel cell death signaling pathways such as those of **necroptosis** and **ferroptosis**. During necroptosis, inflammatory stimuli such as activation of Toll-like receptor (TLR) 4 or TNF receptor 1 signaling activate receptor-interacting protein kinase (RIPK) 1, leading to necrosis by plasma membrane disruption and cell lysis [99].Ferroptosis is driven by iron-dependent phospholipid peroxidation and regulated by several cellular metabolic pathways, including redox homeostasis, iron handling, mitochondrial activity and the metabolism of amino acids, lipids and sugars, as well as various signaling pathways relevant to disease [100]. Necroptosis and ferroptosis have been linked to neurodegeneration in PMS [101,102] as well as other neurological diseases such as Alzheimer’s and Parkinson’s [99,103]. In addition, necroptosis and ferroptosis have been shown to contribute to the formation of neurotoxic microglia and astrocytes [101,104] and reduce the differentiation of OPCs and the ability to myelinate [101,105]. Attempts to inhibit necroptosis and ferroptosis with RIPK inhibitors or ferrostatin-1 analogs reduced neuronal loss and ameliorated disease progression in EAE [101,102], also suggesting positive effects on PMS. 

**Fibrinogen** is a pleiotropic protein, which has essential roles in coagulation, inflammation and tissue repair [106]. If the BBB is disrupted, fibrinogen can enter the CNS and be converted into fibrin, which leads to the activation of immune responses [107]. Fibrin can be detected in the cortex of active but also chronic lesions of progressive MS, and fibrin deposition has been shown to promote neuroinflammation as well as glial scar formation through direct effects on microglia, astrocytes and neurons [108,109,110], resulting in decreased neuronal density [111]. Recently, a monoclonal antibody (5B8) was developed that specifically targets the cryptic fibrin epitope γ377–395 and selectively inhibits fibrin-induced inflammation and oxidative stress without interfering with clotting [112]. In several MS models, 5B8 entered the CNS and bound to parenchymal fibrin, resulting in reduced innate immune response, oxidative stress, demyelination, axonal damage and clinical severity. In PMS, however, the BBB is largely intact, which prevents antibodies from entering the CNS. This could be remedied by the use of shuttles that smuggle the drug into the brain or the fundamental development of new types of drugs with suitable chemical properties that effectively overcome the BBB. 

Targeting the **complement pathway** is another treatment approach that may be useful in PMS. The complement system plays a crucial role in innate immunity and consists of a network of circulating and membrane-expressed proteins that recognize non-self-components [113,114]. After activation via the classical pathway, triggered by antigen-antibody complexes or alternative or lectin pathways, the complement system is critical for the clearance of pathogens as well as apoptotic and dead cells [115]. Several studies have shown that most complement components can also be produced in the CNS [116] and are upregulated in the more severely diseased MS brain [117,118,119,120], suggesting a crucial pathophysiological role in PMS. In EAE, the targeted inhibition of complement activation with complement receptor 2-conjugated inhibitors improved both the acute and chronic stages of disease [121]. Furthermore, the inhibition of the alternative complement pathway with a monoclonal antibody specific for complement factor B attenuated the chronic phase of disease, resulting in less inflammation and demyelination [122]. In addition, treatment with a monoclonal antibody that inhibits complement component (C)1q decreased the concentration of “inflamed” microglia in the hippocampi of mice during chronic EAE, suggesting that complements may play an important role in promoting an inflammatory environment [120]. However, complement components may also have neuroprotective and/or anti-inflammatory properties, as C1q increased the phagocytosis of apoptotic neurons by microglia, resulting in a lower release level of toxic intracellular contents and excitotoxic damage to neighboring neurons [123]. A recent study in PPMS patients found an association between an increased serum C3a/C3 ratio and a higher risk of disability progression, while increased CSF C1q levels tended to be associated with a lower risk of disability progression, suggesting that complement cascades may play a dual role in MS progression [124]. Due to the small number of patients studied, further research is needed to clarify a possible relationship between the complement system and disability progression and to determine whether targeting complement cascades could be a promising treatment strategy for PMS.

## 6. Conclusions

Several therapies effectively target the peripheral immune mechanisms involved in MS relapse biology. Nevertheless, there is a large unmet need in MS for therapies that halt the chronic progression of neurological disability. There is a lot of evidence to suggest that relapsing activity and disease progression are two distinct pathological mechanisms that also require specific, targeted treatment strategies. This could be achieved by new treatment targets that promote neuroprotection and/or remyelination, inhibit cell death signaling pathways and induce the inactivation of fibrinogen and the complement system. In addition, sequential therapy, in which individual drugs are used at certain stages of the disease, or combination therapy, in which therapeutics are used in parallel to treat relapses and disease progression simultaneously, could be a promising approach. The use of a combination therapy could also have positive additive or synergistic effects on disease severity. Recently, the BTK inhibitor tolebrutinib has shown promising results in a phase III trial in PMS and may be the first therapy of its kind to offer a clinically meaningful reduction in disability progression. The identification of further effective therapies for PMS could be supported by novel high-throughput in vitro assays and animal models that better reflect the key clinical features of PMS. In addition, there is an urgent need for reliable biomarkers to more accurately assess treatment response in clinical trials of progressive MS.

## Figures and Tables

**Table 1:** Selection of clinical trials on PMS.

Drug/ Reference	Disease Course/ Sample size	Treatment Duration	Prim. Endpoint	Research Stage/ Status
Tolebrutinib/ HERCULES, NCT04411641	nrSPMS/ N = 1131	24–48 months	6-month CDP	Phase III/ completed
Tolebrutinib/ PERSEUS, NCT04458051	PPMS/ N = 766	12–60 months	3-month cCDP	Phase III/ active, not recruiting
Fenebrutinib/ FENtrepid, NCT04544449	PPMS/ N = 985	120 weeks	Time to Onset of cCDP12	Phase III/ active, not recruiting
Metformin/ NCT05349474	PPMS or SPMS/ N = 44	12 months	Number of adverse events, laboratory abnormalities and new T2 lesions	Phase I/ recruiting
Metformin/ NCT05893225	naPPMS or naSPMS/ N = 120	96 weeks	Change in walking speed as measured by T25FW	Phase II/ recruiting
Metformin/ OCTOPUS, ISRCTN14048364	PPMS or SPMS/ N = 1200	Stage 1: 2 years; stage 2: continued for up to 5 years	Stage 1: whole brain atrophy; stage 2: time to CDP (EDSS, T25FW and 9HPT)	Phase III/ recruiting
Ibudilast/ SPRINT-MS, NCT01982942	PPMS or SPMS/ N = 255	96 weeks	Covariate-adjusted mean rate of change in brain atrophy as measured by BPF	Phase II/ completed
Vidofludimus calcium/ CALLIPER, NCT05054140	PPMS or SPMS/ N = 450	120 weeks	Annualized rate of PBVC during main treatment period	Phase II/ active, not recruiting
Hydroxychloroquine/NCT02913157	PPMS/ N = 35	18 months	T25FW	Phase II/ completed

nrSPMS = nonrelapsing secondary progressive multiple sclerosis; CDP = confirmed disability progression; cCDP = composite confirmed disability progression; cCDP12 = composite 12-week confirmed disability progression; naPPMS = nonactive primary progressive multiple sclerosis; naSPMS = nonactive secondary progressive multiple sclerosis; T25FW = Timed 25-Foot Walk; 9HPT = 9-hole peg test; BPF = Brain Parenchymal Fraction; PBVC = Percent Brain Volume Change.

## Data Availability

No new data were created or analyzed in this study. Data sharing is not applicable to this article.
